# 1990–2021年中国伯基特淋巴瘤疾病负担分析

**DOI:** 10.3760/cma.j.cn121090-20250813-00381

**Published:** 2026-03

**Authors:** 超豪 郑, 钥 王, 婕 陈, 金斗 郭, 金蕾 齐, 脉耕 周, 玉琴 宋, 军 朱, 卫平 刘

**Affiliations:** 1 恶性肿瘤发病机制及转化研究教育部重点实验室，北京大学肿瘤医院暨北京市肿瘤防治研究所淋巴肿瘤内科，北京 100142 Key Laboratory of Carcinogenesis and Translational Research（Ministry of Education / Beijing）, Department of Lymphoma, Peking University Cancer Hospital & Institute, Beijing 100142, China; 2 中国疾病预防控制中心慢性非传染性疾病预防控制中心，北京 100050 Chronic Non-communicable Disease Prevention and Control Center, Chinese Center for Disease Control and Prevention, Beijing 100050, China

**Keywords:** 伯基特淋巴瘤, 疾病负担, 伤残调整寿命年, 发病, 死亡, Burkitt lymphoma, Burden of disease, Disability-adjusted life years, Incidence, Death

## Abstract

**目的:**

分析1990–2021年中国伯基特淋巴瘤（BL）的疾病负担和变化趋势。

**方法:**

基于2021年全球疾病负担研究数据，按照年龄、性别、地区分层，通过BL发病率、死亡率、伤残调整寿命年（DALY）分析疾病负担情况。采用joinpoint软件开展回归分析，计算年龄标准化率的平均年度变化百分比（AAPC）分析变化趋势，并与全球数据进行比较。

**结果:**

2021年，中国BL新发病例1 322例，年龄标准化发病率为0.08/10万；死亡病例241例，年龄标准化死亡率为0.01/10万；DALY为9 516人年，年龄标准化DALY率为0.68/10万。BL疾病负担在不同年龄和性别人群中存在差异，80岁以上年龄组的BL疾病负担最高，发病率、死亡率和DALY率分别为0.60/10万、0.11/10万和1.59/10万；男性BL疾病负担高于女性，男性和女性的年龄标准化发病率分别为0.11/10万和0.05/10万，年龄标准化死亡率分别为0.02/10万和0.01/10万，年龄标准化DALY率分别为0.92/10万和0.43/10万。BL疾病负担存在明显的地区差异，年龄标准化DALY率位于前列的省（自治区、直辖市）为上海市［1.78（95％ *CI*：0.16～3.63）/10万］、西藏自治区［1.15（95％ *CI*：0.41～2.23）/10万］、青海省［1.06（95％ *CI*：0.49～1.87）/10万］；位于后列的为澳门特别行政区［0.32（95％ *CI*：0.07～0.57）/10万］、重庆市［0.36（95％ *CI*：0.14～0.72）/10万］、宁夏回族自治区［0.48（95％ *CI*：0.25～0.92）/10万］。1990–2021年，中国BL年龄标准化发病率增加了187.12％，年龄标准化死亡率和年龄标准化DALY率则分别降低了37.72％和52.70％，AAPC分别为3.49％、−1.46％、−2.40％。

**结论:**

中国BL疾病负担在一定程度上有所减轻，但在不同年龄、性别和地区之间均存在差异，应因地制宜地针对不同人群制定综合防控策略，以期进一步降低BL疾病负担。

## Abstract (en)

**Objective:**

This study aimed to analyze the disease burden and trends of Burkitt lymphoma（BL）in China between 1990 and 2021.

**Methods:**

Based on the 2021 Global Burden of Disease Study data, stratified by age, sex, and region, the disease burden was analyzed by BL incidence, mortality, and disability-adjusted life year（DALY）. Regression analysis was conducted with joinpoint software to calculate the average annual percentage change（AAPC）of age-standardized rates to analyze the changing trends and compare them with global data.

**Results:**

In 2021, a total of 1,322 new BL cases were reported in China, with an age-standardized incidence rate of 0.08/100,000. Of these patients, 241 died, with an age-standardized mortality rate of 0.01/100,000. DALY was 9,516 person-years, with an age-standardized DALY rate of 0.68/100,000. The disease burden of BL demonstrated significant differences across various age and sex groups. The highest BL disease burden was observed in the age group over 80 years old, with an incidence of 0.60/100,000, mortality of 0.11/100,000, and DALY rates of 1.59/100,000. BL disease burden was higher in males than in females, with age-standardized incidence rates of 0.11/100,000 and 0.05/100,000, age-standardized mortality rates of 0.02/100,000 and 0.01/100,000, and age-standardized DALY rates of 0.92/100,000 and 0.43/100,000, respectively. Significant regional differences were observed in BL disease burden. The highest-ranked age-standardized DALY rates were reported in Shanghai［1.78（95％ *CI*: 0.16–3.63）/100,000］, Tibet Autonomous Region［1.15（95％ *CI*: 0.41–2.23）/100,000］and Qinghai Province［1.06（95％ *CI*: 0.49–1.87）/100,000］, whereas the lowest-ranked were observed in Macao Special Administrative Region［0.32（95％ *CI*: 0.07–0.57）/100,000］, Chongqing［0.36（95％ *CI*: 0.14–0.72）/100,000］and Ningxia Hui Autonomous Region［0.48（95％ *CI*: 0.25–0.92）/100,000］. From 1990 to 2021, the age-standardized incidence rate of BL increased by 187.12％, whereas the age-standardized mortality rate and age-standardized DALY rate decreased by 37.72％ and 52.70％, respectively. The AAPCs were 3.49％, −1.46％, and −2.40％, respectively.

**Conclusion:**

The disease burden of BL in China has been alleviated to a certain extent; however, significant differences were observed among different ages, genders, and regions. Comprehensive prevention and control strategies should be formulated according to local conditions for different populations to further reduce the disease burden.

伯基特淋巴瘤（BL）是一种高度侵袭性、生长迅速的B细胞非霍奇金淋巴瘤（NHL），常累及颌骨、中枢神经系统、结直肠、肾脏或其他器官及部位[1]。BL有3种组织学上难以区分的病因亚型：地方流行型、散发型和免疫缺陷相关型[2]。地方流行型BL是撒哈拉以南非洲最常见的儿童恶性肿瘤，通常表现为颌骨或眼眶肿瘤，与恶性疟原虫和EB病毒（EBV）感染有关[3]。散发型BL没有特定的地理分布，往往发生于肠道的淋巴组织，以腹部肿瘤为特征[4]。免疫缺陷相关型BL多见于人类免疫缺陷病毒（HIV）感染[5]或有器官移植史的患者[6]。2021年全球疾病负担（GBD2021）研究估计2021年全球BL新发病例19 072例，死亡病例6 525例，伤残调整寿命年（DALY）为392 126人年[7]。全球BL年龄标准化发病率、死亡率和DALY率均呈上升趋势，分别以年均2.05％、0.56％、0.44％的速率上升。本研究基于GBD2021数据，对1990–2021年中国BL的疾病负担状况及变化趋势进行分析，旨在为中国BL的预防和控制提供参考依据。

## 资料与方法

1. 资料来源：BL流行病学数据来源于GBD2021数据库（https://vizhub.healthdata.org/gbd-results/）。该数据库是一个综合数据库，提供了204个国家和地区的371种疾病和88种可归因风险因素的流行病学数据[7]。本研究通过GHDx查询工具提取了1990–2021年中国大陆（未包含中国台湾省数据）及全球BL发病、死亡和DALY等相关数据，人群按照性别和年龄分组（从<5岁至≥80岁，每5年1个间隔，共17个年龄组）提取数据。根据国际疾病分类第10版（ICD-10）编码，BL编码为C83-C83.7[8]。

2. 评价指标：本研究基于2021年全球疾病负担研究数据，按照年龄、性别、地区分层，通过BL发病率、死亡率、DALY及其年龄标准化率（ASR）分析疾病负担情况。ASR是按照GBD世界标准人口的年龄构成，对研究人群的粗率（如发病率、死亡率）进行调整后得到的率，计算公式为$ASR=\frac{\sum_{i=1}^{A}a_{i}w_{i}}{\sum_{i=1}^{A}w_{i}}\times 100 000$[9]。DALY是以时间为单位综合衡量死亡和伤残所造成的健康损失，为早死损失健康寿命年（YLL）和伤残损失健康寿命年（YLD）之和[10]。

3. 统计学处理：本研究采用R4.5.2软件，对1990–2021年中国（未包含中国台湾省数据）及全球BL患者的发病、死亡、DALY等相关数据进行分析整理。采用Joinpoint Regression Program 5.4.0软件对上述数据进行变化趋势分析，通过对数线性回归模型计算ASR的平均年度变化百分比（AAPC）及95％置信区间（*CI*）以分析疾病负担变化趋势[11]，并与全球数据进行比较，*P*<0.05为差异具有统计学意义。

## 结果

1. 2021年不同性别及年龄组BL疾病负担：2021年，中国BL新发病例1 322例，年龄标准化发病率为0.08/10万；死亡病例241例，年龄标准化死亡率为0.01/10万；DALY为9 516人年，年龄标准化DALY率为0.68/10万。中国BL发病率、死亡率和DALY率随年龄增长呈双峰状分布，即先上升后下降再上升，约65岁后迅速上升。65岁以上人群发病人数和死亡人数分别约占总人群的41％和44％。中国新发病例和死亡病例最大值均在80岁以上年龄组，最大值分别为198例和38例，DALY最大值在5～9岁年龄组，最大值为1 294人年，发病率、死亡率和DALY率最大值均在80岁以上年龄组，最大值分别为0.60/10万、0.11/10万和1.59/10万（图1）。

**图1:**
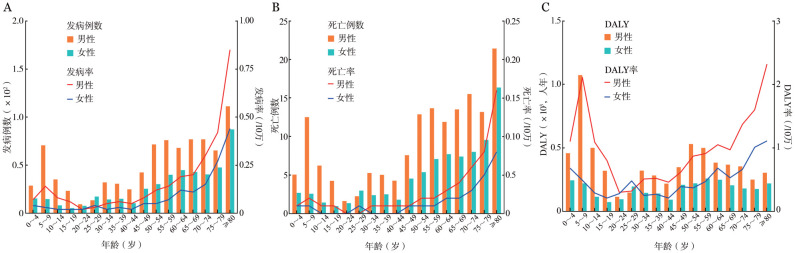
2021年不同年龄组男性和女性伯基特淋巴瘤发病、死亡、伤残调整寿命年（DALY）情况 **A** 不同年龄组男性和女性发病情况；**B** 不同年龄组男性和女性伯基特淋巴瘤患者死亡情况；**C** 不同年龄组男性和女性伯基特淋巴瘤患者DALY情况

男性BL疾病负担高于女性。男性和女性的年龄标准化发病率分别为0.11/10万和0.05/10万，年龄标准化死亡率分别为0.02/10万和0.01/10万，年龄标准化DALY率分别为0.92/10万和0.43/10万。男性和女性发病率、死亡率和DALY率最高值均在80岁以上年龄组，男性80岁以上年龄组发病率、死亡率和DALY率分别为0.85/10万、0.16/10万和2.32/10万；女性80岁以上年龄组发病率、死亡率和DALY率分别为0.44/10万、0.08/10万和1.11/10万（图1）。

2. 2021年全国各省（自治区、直辖市）BL疾病负担：全国各省（自治区、直辖市）的BL疾病负担存在明显的地区差异。年龄标准化发病率位于前列的省（自治区、直辖市）为上海市［0.31（95％ *CI*：0.02～0.69）/10万］、北京市［0.20（95％ *CI*：0.04～0.40）/10万］、广东省［0.12（95％ *CI*：0.04～0.22）/10万］，位于后列的为西藏自治区［0.03（95％ *CI*：0.01～0.05）/10万］、云南省［0.03（95％ *CI*：0.02～0.05）/10万］、重庆市［0.04（95％ *CI*：0.01～0.07）/10万］；年龄标准化死亡率位于前列的省（自治区、直辖市）为上海市［0.04（95％ *CI*：0～0.08）/10万］、北京市［0.02（95％ *CI*：0～0.05）/10万］、青海省［0.02（95％ *CI*：0.01～0.04）/10万］，位于后列的为澳门特别行政区［0.01（95％ *CI*：0～0.01）/10万］、重庆市［0.01（95％ *CI*：0～0.01）/10万］、河南省［0.01（95％ *CI*：0～0.02）/10万］；年龄标准化DALY率位于前列的省（自治区、直辖市）为上海市［1.78（95％ *CI*：0.16～3.63）/10万］、西藏自治区［1.15（95％ *CI*：0.41～2.23）/10万］、青海省［1.06（95％ *CI*：0.49～1.87）/10万］，位于后列的为澳门特别行政区［0.32（95％ *CI*：0.07～0.57）/10万］、重庆市［0.36（95％ *CI*：0.14～0.72）/10万］、宁夏回族自治区［0.48（95％ *CI*：0.25～0.92）/10万］。

3. 1990–2021年BL疾病负担变化趋势分析：1990–2021年，中国BL年龄标准化发病率从0.03/10万增至0.08/10万，增幅为187.12％；年龄标准化死亡率从0.02/10万降至0.01/10万，降幅为37.72％；年龄标准化DALY率从1.44/10万降至0.68/10万，降幅为52.70％。中国BL年龄标准化发病率以3.49％的AAPC上升，上升速率高于全球水平，而年龄标准化死亡率和年龄标准化DALY率与全球变化趋势相反，分别以1.46％、2.40％的AAPC下降。男性年龄标准化发病率、死亡率和DALY率变化幅度分别为219.52％，−30.61％、−45.31％，女性则分别为137.01％、−48.79％、−64.37％（图2，表1）。

**图2:**
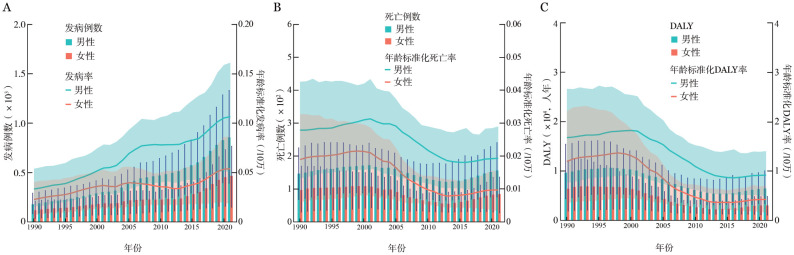
1990–2021年中国伯基特淋巴瘤男性和女性患者疾病负担变化趋势 **A** 1990–2021年男性和女性发病情况；**B** 1990–2021年男性和女性伯基特淋巴瘤患者死亡情况；**C** 1990–2021年男性和女性伯基特淋巴瘤患者DALY情况 **注** DALY：伤残调整寿命年

**表1:** 1990–2021年中国和全球伯基特淋巴瘤的ASR平均年度变化百分比（AAPC）

国家和地区	性别	年龄标准化发病率			年龄标准化死亡率			年龄标准化DALY率		
		AAPC^a^	95％ *CI*	*P*值	AAPC^a^	95％ *CI*	*P*值	AAPC^a^	95％ *CI*	*P*值
中国	总体	3.49	（2.81～4.17）	<0.001	−1.46	（−1.77～−1.15）	<0.001	−2.40	（−2.68～−2.12）	<0.001
	男性	3.90	（3.35～4.45）	<0.001	−1.14	（−1.34～−0.94）	<0.001	−1.89	（−2.12～−1.67）	<0.001
	女性	2.95	（2.53～3.38）	<0.001	−2.11	（−2.47～−1.75）	<0.001	−3.26	（−3.80～−2.72）	<0.001
全球	总体	2.05	（1.94～2.16）	<0.001	0.56	（0.50～0.62）	<0.001	0.44	（0.34～0.54）	<0.001
	男性	2.07	（1.87～2.27）	<0.001	0.55	（0.48～0.63）	<0.001	0.44	（0.37～0.51）	<0.001
	女性	2.01	（1.84～2.19）	<0.001	0.56	（0.40～0.72）	<0.001	0.42	（0.29～0.56）	<0.001

**注** ASR：年龄标准化率；DALY：伤残调整寿命年；^a^AAPC明显不等于0，即*P*<0.05，差异有统计学意义

## 讨论

GBD研究的中国数据来自中国疾病监测点系统、中国疾病预防控制中心死因报告系统和肿瘤登记处等，通过模型拟合等方式得出特定疾病的负担情况，与中国权威机构发布的疾病负担数据具有较高的一致性，为系统量化健康损失提供了关键依据[10]。如国家癌症中心研究[12]预计2016年中国淋巴瘤和骨髓瘤新发病例89 900例，死亡病例51 500例；GBD2016研究[13]–[14]预计同年新发病例91 900例（其中淋巴瘤75 400例、骨髓瘤16 500例），死亡病例50 800例（其中淋巴瘤40 500例、骨髓瘤10 300例）。一项整合了国家癌症中心、中国儿童白血病登记库、中国成人白血病登记库、医院质量检测系统和死因报告系统的研究[15]估计2019年中国急性白血病新发病例43 275例，死亡病例27 049例，该结果与GBD2019[16]估算的数据基本吻合（新发54 688例，死亡31 918例）。

本研究基于GBD2021数据发现，中国BL疾病负担随年龄增长呈双峰状分布，男性疾病负担高于女性，且不同地区之间存在显著差异；过去30年间，尽管中国BL年龄标准化发病率增加了1.8倍，但是死亡率下降了1/3，DALY率下降了一半，反映了中国的BL防治工作卓有成效。

BL疾病负担与EBV感染、人口老龄化、生活方式的改变等因素有关。一项基于GLOBOCAN2018数据库的研究估计，2018年全球约6 600例新发BL病例与EBV有关，占全球新发BL病例的55％[17]。不同BL亚型与EBV的相关性存在差异，95％～100％的地方流行型BL病例与EBV感染有关，20％～30％散发型BL病例与EBV感染有关，25％～40％的免疫缺陷相关型BL病例与EBV感染有关[2]。一项基于GBD2021数据库的研究发现，全球BL发病率、死亡率、患病率、DALY变化归因于人口老龄化的占比分别为13.04％、−6.39％、9.53％、−39.89％[18]。中国预期老龄化率将从2020年的11.97％上升到2044年的24.71％[19]，随着中国老龄化情况日趋严重，中国BL疾病负担也将发生显著变化。此外，BL疾病负担变化部分可归因于生活方式的变化以及逐渐增高的体重指数（BMI）[20]。一项全国性调查共纳入645 223名18～69岁成年人，结果显示中国标准化平均BMI从2004年的22.7 kg/m^2^增加到2018年的24.4 kg/m^2^［21]，而中国BL的DALY的0.02％与BMI相关[22]。因此，亟待针对BL危险因素开展更加精准的防控措施以降低BL的疾病负担。

中国BL疾病负担在不同年龄和性别人群中存在差异。本研究发现BL发病率在0～14岁儿童和65岁以上人群中呈现双峰状分布。0～14岁儿童较高的BL发病率可能与中国儿童EBV感染的流行有关。一项来自上海的回顾性分析纳入10 260例接受EBV核酸检测的住院患儿，结果发现EBV阳性率为21.4％，平均年龄为7.3岁[23]。65岁以上人群也有较高的BL发病率，这可能与老年人免疫系统的衰老有关，常表现为对感染、癌症和自身免疫性疾病易感性增加[24]。此外，男性BL疾病负担在大部分年龄组中均高于女性，这可能与男性特有的遗传易感性及男女生活方式的差异有关[22],[25]。

总体上看，中国BL疾病负担整体呈现下降趋势。尽管中国BL发病率呈现上升趋势，但死亡率呈现下降趋势。发病率表现为东部较高，中部次之，西部最低，这可能与各省（自治区、直辖市）居民的健康意识和医疗资源可及性有关[26]。死亡率的下降则可能与基本医疗保险覆盖率越来越高、诊断技术的进步和治疗策略的优化等因素有关[27]。低社会人口指数的地区往往有较高的淋巴瘤疾病负担，而高社会人口指数的地区往往有较低的淋巴瘤疾病负担[28]。从整体上看，中国东部地区的BL疾病负担低于中西部地区，这可能与经济发展水平和医疗资源可及性等因素有关[26]。但也有例外，如上海市BL的年龄标准化DALY率是其他地区的2～3倍。一方面，这可能与上海市居民较高的预期寿命有关，2019年上海市男性和女性的预期寿命分别为80.4岁和85.5岁，明显高于其他省（自治区、直辖市）[29]；另一方面，在过去数十年里，上海市经历了人口老龄化，经济发展和社会转型的快速变化，生活方式的改变、居民肥胖率的持续上升等都是DALY增加的重要原因[30]。

本研究存在一定的局限性：本研究的数据来自GBD2021数据库，采用的是拟合后的数据，可能与现况调查数据存在偏倚；中国尚无全国层面的EBV流行病学数据，EBV与BL疾病负担的关系仍然需要进一步深入研究；近30年来BL诊断技术和治疗水平有所提升，诊断检出率的提高以及治疗预后的改善对疾病负担的影响需要进一步在研究中进行探讨；此外，由于每个亚组的样本量较小，本研究未能进一步分析城镇和农村BL疾病负担的差异。

综上所述，过去30年间，中国BL疾病负担发生显著改变，尽管年龄标准化发病率呈上升趋势，但是年龄标准化死亡率和年龄标准化DALY率呈下降趋势，疾病负担在一定程度上有所减轻。另一方面，中国BL疾病负担在不同年龄、性别和地区之间均存在显著差异，应因地制宜地针对不同人群制定综合防控策略，以期进一步降低BL疾病负担。
